# Waist-to-height ratio for OSA risk: a comparative analysis of NHANES and a clinical data

**DOI:** 10.3389/fmed.2026.1842979

**Published:** 2026-05-29

**Authors:** Yajing Li, Jingjing Zhang, Xiaoyun Zhao, Yuechuan Li

**Affiliations:** Department of Respiratory and Critical Care Medicine, Chest Hospital, Tianjin University, Tianjin, China

**Keywords:** BMI, NHANES (National Health and Nutrition Examination Survey), waist-to-height ratio, waist circumference, obstructive sleep apnea

## Abstract

**Objective:**

This study aims to demonstrate the relationship between waist-to-height ratio (WHtR) and obstructive sleep apnea (OSA) risk by analyzing data from the National Health and Nutrition Examination Survey (NHANES) and clinical cases.

**Methods:**

This study utilized data from the National Health and Nutrition Examination Survey (NHANES), a nationally representative cross-sectional survey of the US non-institutionalized population, and a retrospective clinical dataset of 200 patients who underwent polysomnography (PSG) at the Sleep Center of Tianjin Chest Hospital, China. Multivariate logistic regression analysis, restricted cubic spline (RCS) analysis, receiver operating characteristic (ROC) curves and subgroup analysis were used to assess the association between WHtR and the OSA risk.

**Results:**

WHtR was positively associated with OSA risk in both datasets. In the adjusted model, each 0.1-unit increase in WHtR was associated with higher odds of OSA (NHANES: OR 2.780, 95% CI: 2.536–3.048; clinical: OR 11.998, 95% CI: 6.010–23.953; both *P* < 0.001). RCS analysis revealed a significant non-linear relationship (*P* for non-linearity <0.001), with risk increasing rapidly beyond thresholds of 0.589 (NHANES) and 0.523 (clinical). ROC analysis showed that WHtR achieved an AUC of 0.727 in NHANES and 0.883 in clinical data, outperforming BMI in the clinical setting. DeLong's test confirmed that WHtR's AUC was significantly larger than that of BMI (Z = 7.186, *P* < 0.001), while no significant difference was detected between WHtR and waist circumference (Z = 0.543, *P* = 0.587) in the clinical data. In subgroup analyses of clinical data, WHtR maintained superior predictive value across sex and age strata (all *P* < 0.001 vs. BMI by DeLong's test).

**Conclusions:**

WHtR is a strong indicator of an increased OSA risk. WHtR has superior predictive value for OSA compared to BMI in clinical data, but shows equivalence in population data. WHtR demonstrates promise as a screening tool in a high-prevalence, clinically referred Chinese population.

## 1 Introduction

Obstructive sleep apnea (OSA) is a common sleep-related breathing disorder, characterized by the collapse and obstruction of the upper airway during sleep, leading to apneic or hypoxic events. It is often manifested as snoring, disrupted sleep architecture, and intermittent hypoxemia. OSA is widely prevalent globally, with an estimated 936 million adults suffering from mild to severe obstructive sleep apnea and 425 million adults suffering from moderate to severe obstructive sleep apnea (1). China is the country with the heaviest burden globally (1, 2). The prevalence of OSA in China has risen significantly from 8.1% during 2000–2005 to 26.9% during 2021–2024 (2). OSA is closely related to the development of cardiovascular diseases, neurological disorders, and metabolic diseases, resulting in high medical costs and a serious social burden. However, public awareness of this disease is generally low. Therefore, screening for high-risk groups of OSA is of great significance for improving the diagnosis and treatment rate and reducing the disease burden of OSA.

Obesity is the most important and modifiable risk factor for OSA. The proportion of obese individuals is increasing continuously on a global scale (3). In obese individuals, fat accumulation in the neck leads to hypertrophy of the soft tissues around the pharynx, which increases the pressure around the pharynx and makes the pharyngeal airway more susceptible to collapse during sleep. Accumulation of fat in the abdomen restricts the movement of the diaphragm, resulting in reduced lung capacity and consequently decreased pharyngeal airway dilating force (4). Moreover, the systemic inflammatory response caused by obesity may affect the structure and function of the upper airway, further exacerbating the symptoms of OSA (5). Data from the Sleep Heart Health Study (SHHS) show that among obese individuals (men with a BMI ≥30.9 and women with a BMI > 31.7), approximately 69% exhibit symptoms of OSA to varying degrees, and about 32% suffer from moderate to severe OSA (6). Obesity has now been recognized as a disease (7, 8). To optimize the clinical assessment of obesity, the new guidelines highlights the limitations of BMI in diagnosing obesity. First, BMI only takes into account the ratio of weight to height and cannot distinguish between fat and muscle, nor can it reflect the distribution of fat (9). It suggests that in addition to BMI, at least one anthropometric criterion (waist circumference, waist-to-hip ratio, or waist-to-height ratio) must also be met (10, 11).

The gold standard for diagnosing OSA relies on the results of polysomnography (PSG). This test requires patients to spend a full night in a hospital sleep monitoring center, where multiple signals are recorded to determine whether the patient experiences sleep apnea during this period. However, due to the high cost of PSG equipment and the stringent requirements for the testing environment, the widespread adoption of this test is relatively limited. Nevertheless, various screening methods are now available for clinical use, such as questionnaire-based screening and portable sleep monitor screening. Whether it is the STOP-Bang questionnaire, the Berlin questionnaire, or the NoSAS score, the scoring criteria of these questionnaires all include BMI. Therefore, identifying more accurate scoring indicators is crucial for the initial screening of OSA. The Waist-to-Height Ratio (WHtR), which is the ratio of waist circumference to height, is an excellent alternative indicator that has emerged to meet this need. It precisely quantifies the degree of central obesity (i.e., abdominal fat accumulation), which is the key factor driving the pathophysiological processes of OSA. Compared to waist circumference alone, WHtR takes into account individual differences in height. A WHtR cutoff of ≥0.5 has been proposed and validated as a universal threshold for central obesity across diverse populations, applicable to both children (age ≥6 years) and adults (12). According to a cross-sectional survey involving 30,630 Chinese adults, a WHtR of 0.5 can be used as the cutoff value for evaluating central obesity in the Chinese population (13). Studies have confirmed that WHtR outperforms BMI in predicting both cardiometabolic risk and OSA (14). This study, based on data from NHANES and clinical sources, investigates the correlation between WHtR and OSA, and determines whether WHtR is superior to traditional indicators such as BMI in diagnosing OSA.

## 2 Methods

### 2.1 Data sources

**Figure 1:**
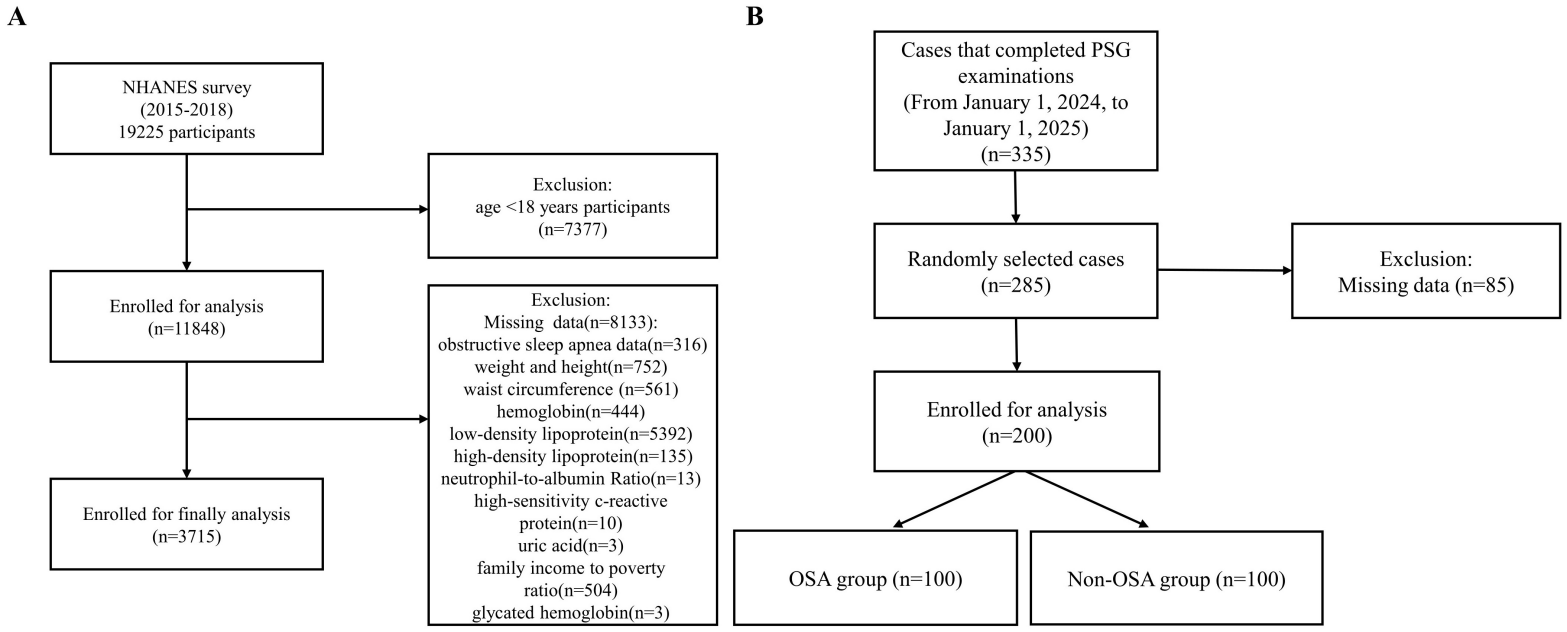
Flowchart of the study population. **(A)** Flowchart for the NHANES data. **(B)** Flowchart for the clinical data.

The data analyzed in this study are publicly available from the NHANES database, a nationally representative, cross-sectional survey conducted by the National Center for Health Statistics (NCHS) at the Centers for Disease Control and Prevention in the United States of America. NHANES employs a complex, multistage probability sampling design to assess the health and nutritional status of the non-institutionalized civilian US population. The survey combines household interviews with standardized physical examinations and laboratory tests conducted in mobile examination centers The NHANES study protocol was approved by the Research Ethics Review Board of the National Center for Health Statistics (NCHS) and written informed consent was obtained from all participants. All research procedures in this study strictly adhered to the Strengthening the Reporting of Observational Studies in Epidemiology (STROBE) guidelines. In this study, we extracted data from the NHANES database from 2015–2018, and a total of 19,225 participants were included. We included demographic characteristics and anthropometric measurements as covariates. We excluded 7,377 participants under the age of 18 and 8,133 participants due to missing data on key variables (including OSA status, waist circumference, height, or weight), yielding a final analytic sample of 3,715 participants (Figure 1A). All analyses incorporated the appropriate sample weights, clustering, and stratification variables to account for the complex survey design and to obtain nationally representative estimates, in accordance with the NHANES analytic guidelines.

All patients in this group were referred for overnight polysomnography (PSG) due to clinically suspected OSA, including symptoms such as habitual snoring, excessive daytime sleepiness, witnessed apneas, or gasping/choking during sleep. This reflects a high pre-test probability clinical setting typical of sleep center referrals. The sample size for the clinical validation group was determined *a priori* based on precision requirements for diagnostic accuracy estimates (15). Assuming an expected sensitivity of 85% and a desired 95% confidence interval width of ±10%, and given that the prevalence of OSA in China is approximately 26.9% (2), the calculated total sample size was 207 cases. Therefore, we collected data from all patients who completed polysomnography (PSG) examinations at the Sleep Center of Tianjin Chest Hospital between January 1, 2024, and January 1, 2025, totaling 335 cases. And then, 285 cases were randomly selected from it. The random selection process was conducted as follows: First, all the 335 cases were assigned a unique sequential number, and 285 cases were selected using a computer-generated random number table (Excel RAND function). This process ensured that each eligible case had an equal probability of selection. The selected cases were not consecutive patients, rather, they were randomly distributed throughout the study period to minimize temporal bias. After excluding 85 patients with incomplete information, 200 cases were finally enrolled in this retrospective study. This retrospective study was approved by the Ethics Committee of Tianjin Chest Hospital (approval number: 2025LW-035) with a waiver of informed consent, as the study used anonymized existing medical records and the patients were not available for re-consent. Figure 1B shows the details of the selection process.

### 2.2 Definition and assessment of OSA and other covariates used in the NHANES

In NHANES, OSA status was determined via self-reported questionnaire data about sleep-related symptoms, not by polysomnography. Participants answer specific questions, and according to relevant studies, OSA is defined by any of the following conditions (16): (1) snoring three or more times per week; (2) gasping, snorting, or experiencing breathing cessation for three or more nights per week; or (3) feeling excessively sleepy during the day at least 16 times, despite sleeping for seven or more hours each night.

Demographic covariates were obtained through self—report questionnaires, including age, sex. Trained health technicians measured the participants' height, weight, and waist circumference and calculated the BMI. WHtR was calculated as waist circumference divided by height.

### 2.3 Clinical assessment and anthropometric measurements

All cases undergoing PSG were from outpatients or inpatients with a suspected diagnosis of OSA. Before PSG, a well-trained pulmonologist specializing in sleep-related respiratory disorders would conduct an interview on basic information, present illness, past medical history and personal history. General physical measurements would be taken in accordance with the recommended procedures and using precise instruments. Height, weight, waist circumference, and neck circumference would be collected using standard methods (17). WHtR would be calculated using the formula (waist circumference/height), and BMI would be calculated using the formula (weight/height^2^) (18).

### 2.4 Polysomnography

In the Sleep Center of Tianjin Chest Hospital, overnight PSG (EMBLA N7000) was conducted to monitor the following physiological variables: electroencephalogram (EEG); electrooculogram (EOG); electrocardiogram (ECG); respiratory inductance plethysmography (RIP) to monitor respiratory effort during thoracoabdominal movements. Airflow was assessed using an oronasal thermal and pressure sensor. Oxygen saturation was recorded using a finger pulse oximeter.

Sleep stages were determined according to the 2017 American Academy of Sleep Medicine (AASM) criteria (19). Apnea was defined as a reduction in airflow of ≥90% relative to the pre-event baseline for ≥10 s. Hypopnea was defined as a reduction in airflow of ≥30% relative to the pre-event baseline for ≥10 s, accompanied by a ≥3% desaturation in oxygen saturation. The third edition of the International Classification of Sleep Disorders (ICSD-3) defines OSA as a PSG-determined obstructive respiratory disturbance index (RDI) ≥5 events/h associated with the typical symptoms of OSA (e.g., unrefreshing sleep, daytime sleepiness, fatigue or insomnia, awakening with a gasping or choking sensation, loud snoring, or witnessed apneas), or an obstructive RDI ≥15 events/h (even in the absence of symptoms) (20).

### 2.5 Statistical analysis

The categorical variables are represented as frequencies or percentages; in contrast, continuous variables are assessed for normality via the Kolmogorov–Smirnov test. The continuous variables are not normally distributed and are instead represented by quartiles. We used the Mann–Whitney U test to analyze continuous variables and the chi–square test for categorical variables, with a focus on the study population's characteristics.

We performed multivariate logistic regression to assess the relationship between the WHtR, waist circumference, BMI and OSA. We evaluated in two models. Model 1 was unadjusted; Model 2 was adjusted for age and gender. The results are shown as ORs along with their corresponding 95% CIs.

Restricted cubic spline (RCS) plots was used to illustrate the nonlinear relationship between WHtR and OSA risk and compared the results before and after adjustment.

To assess the value of WHtR in predicting the risk of OSA, we conducted a comparative analysis using receiver operating characteristic (ROC) curves. We compared the predictive performance of WHtR, waist circumference, and BMI in terms of their ability to predict OSA risk. The area under the ROC curve (AUC) was calculated for each measure to evaluate and compare their predictive accuracy. Pairwise comparisons of AUCs between different anthropometric indicators were performed using DeLong's test, which accounts for the correlated nature of ROC curves derived from the same sample. Statistical significance was set at two-sided P <0.05.

To assess the robustness of our findings, we performed sensitivity analyses within the clinical data. Subgroup analyses were conducted stratified by sex (male, female) and age (age ≥60; age <60). Within each subgroup, ROC curve analyses were performed for WHtR, waist circumference, and BMI. The AUCs were compared pairwise using DeLong's test to determine if the predictive superiority of WHtR was consistent across these subgroups.

Descriptive and correlation analyses were conducted in accordance with the NHANES statistical analysis protocol to obtain nationally representative data (21). In the analysis, we considered the study sampling design, data clustering, and subsample weights. All the statistical analyses were conducted via R (version 4.4.1) and IBM SPSS Statistics (version 27.0.1). Two-sided *P* values <0.05 were considered statistically significant in this study.

## 3 Results

### 3.1 Population characteristics of the study subjects in NHANES and clinical data

Based on the analysis of NHANES data, compared with the non-OSA group, the OSA group is older, with significant increases in weight, BMI, waist circumference, and WHtR, while there is no significant difference in height. The non-OSA group consists predominantly of females. A retrospective analysis of the collected clinical cases indicates that there are no significant differences between the two groups in terms of gender, weight, and height. The OSA group has significantly higher waist circumference, BMI, and WHtR compared with the non-OSA group. Tables 1, 2 shows the characteristics of the study participants.

**Table 1:** Population characteristics of study subjects in NHANES.

Variable	OSA (*n* = 1,707)	non-OSA (*n* = 2,008)	Statistics	*P* value
Age (years)	54.00 (40.00, 65.00)	45.00 (29.00, 63.00)	−9.719	<0.001^*^
Gender			23.335	<0.001^‡^
Male	911 (50.00%)	912 (50.00%)		
Female	796 (42.10%)	1,096 (57.90%)		
Race				
Mexican American	309 (18.10%)	260 (12.95%)	28.709	<0.001^‡^
Other Hispanic	215 (12.60%)	213 (10.61%)		
Non-Hispanic White	568 (33.27%)	741 (36.90%)		
Non-Hispanic Black	349 (20.45%)	413 (20.57%)		
Other race	266 (15.58%)	381 (18.97%)		
Weight (kg)	86.00 (73.90, 102.40)	72.30 (61.60, 85.10)	−21.919	<0.001^*^
Height (cm)	166.90 (159.70, 173.90)	166.20 (159.00, 173.80)	−1.826	0.068^*^
BMI (kg/cm^2^)	30.80 (27.10, 35.70)	26.20 (22.90, 29.90)	−23.987	<0.001^*^
Waist circumference (cm)	104.70 (95.70, 116.30)	93.00 (82.50, 103.00)	−24.824	<0.001^*^
Waist-to-height ratio	0.62 (0.57,0.69)	0.56 (0.49,0.62)	−23.882	<0.001^*^

OSA, Obstructive sleep apnea. ^*^Mann–Whitney U test for continuous variables. ^‡^Pearson's chi-squared test for categorical variables.

**Table 2:** Population characteristics of study subjects in clinical data.

Variable	OSA (*n =* 100)	non-OSA (*n* = 100)	Statistics	*P* value
Age (years)	62.00 (51.00, 69.00)	49.00 (39.25, 64.00)	−4.293	<0.001^*^
Gender			0.187	0.666^‡^
Male	61 (61.00%)	58 (58.00%)		
Female	39 (39.00%)	42 (42.00%)		
Weight (Kg)	80.00 (67.25, 90.00)	75.00 (62.62, 85.75)	−1.453	0.416^*^
Height (cm)	169.00 (60.00, 174.00)	170.00 (162.25, 175.00)	−0.953	0.341^*^
Neck circumference (cm)	39.00 (37.00, 42.00)	38.00 (31.00, 41.00)	−3.086	0.002^*^
Waist circumference (cm)	95.50 (90.00, 100.00)	79.00 (75.40, 85.00)	−8.869	<0.001^*^
BMI (Kg/m^2^)	27.25 (24.52, 30.80)	26.00 (23.02, 30.04)	−2.159	0.031^*^
Waist-to-height ratio	0.56 (0.53, 0.61)	0.46 (0.45, 0.48)	−9.353	<0.001^*^

OSA, Obstructive sleep apnea. ^*^Mann–Whitney U test for continuous variables. ^‡^Pearson's chi-squared test for categorical variables.

### 3.2 Observational associations between the WHtR, Waist circumference, BMI and OSA in NHANES and clinical data

**Figure 2:**
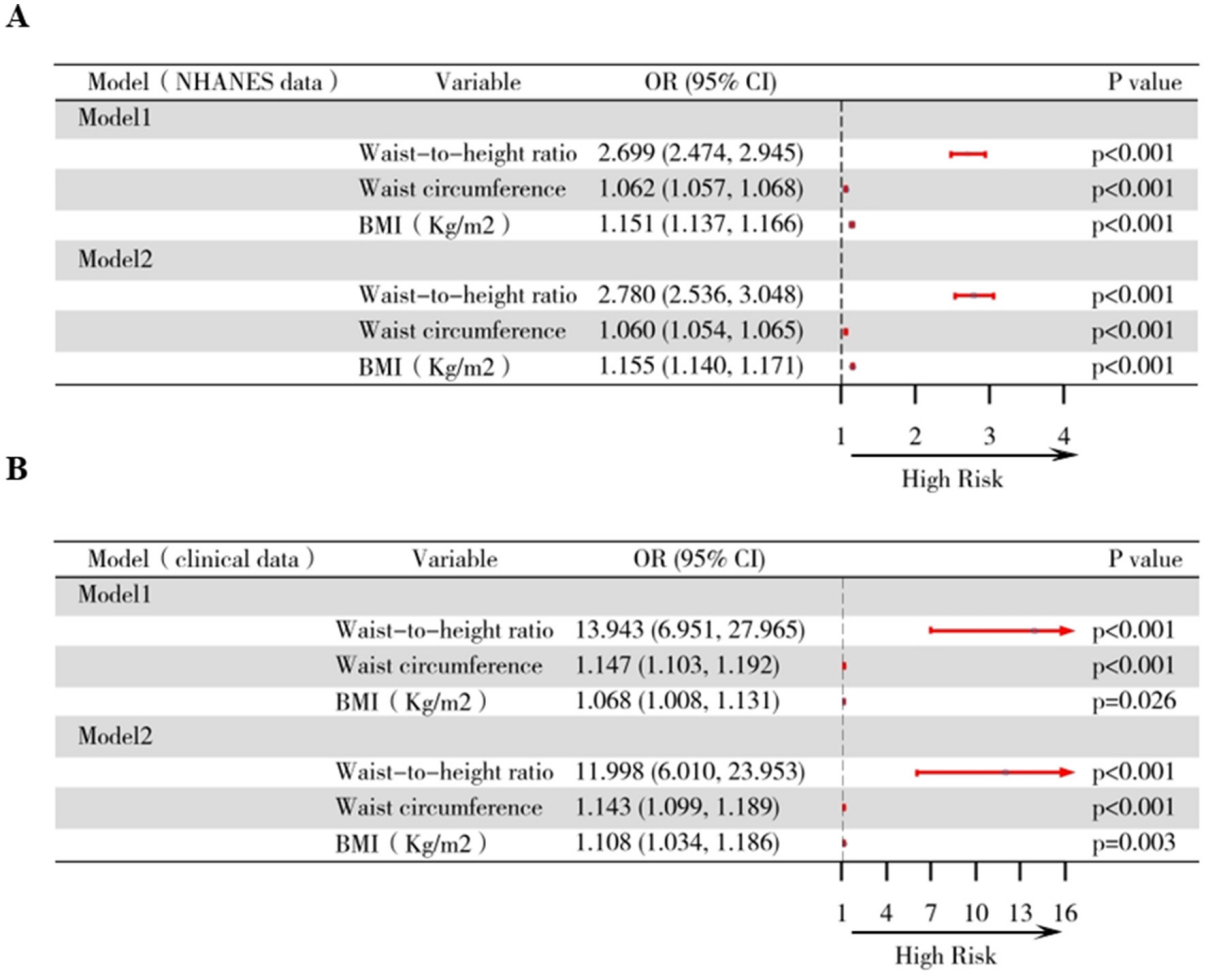
Forest plot of logistic regression analyses for different models, with waist-to-height ratio, waist circumference, and BMI as predictors and OSA as the outcome**. (A)** Forest plot of logistic regression analyses in the NHANES data. **(B)** Forest plot of logistic regression analyses in the clinical data. OR, odds ratio; CI, confidence interval; WHtR, waist-to-height ratio. Model 1 was unadjusted; Model 2 was adjusted for age and gender.

Logistic regression analysis showed a significant positive association between WHtR, waist circumference, BMI and the odds of OSA in unadjusted and adjusted models based on the NHANES and clinical data (Figure 2). WHtR exhibits the strongest association with OSA. In the adjusted model, this association remained reliable robust (OR per 0.1 unit increase, 2.780; 95% CI:2.536, 3.048, *p* < 0.001), meaning that each 0.1 unit increase in WHtR was associated with 178% higher odds of OSA in NHANES data, while in clinical data, this association remained more robust, each 0.1 unit increase in WHtR was associated with 1,099% higher odds of OSA (OR per 0.1 unit increase, 11.998; 95% CI:6.010, 23.953, *p* < 0.001).For clinical interpretability, we also report that each 0.01 unit increase in WHtR (equivalent to 1 cm increase in waist circumference for a 170 cm tall individual) was associated with 28.2% higher odds of OSA (OR 1.282; 95% CI: 1.196, 1.374, *p* < 0.001).

**Figure 3:**
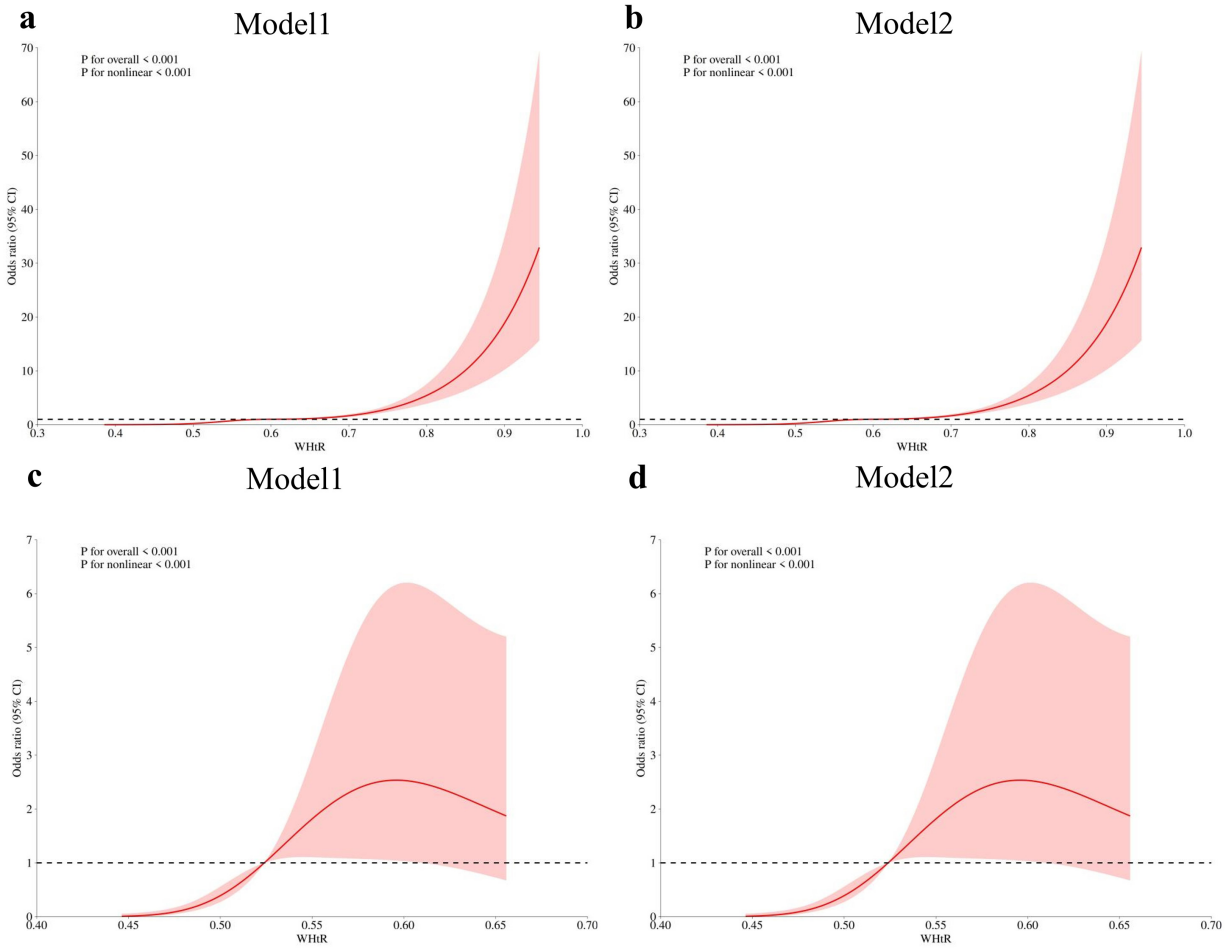
Non-linear relationship between the WHtR and OSA. OR, odds ratio; CI, confidence interval; WHtR, waist-to-height ratio. Model 1 was unadjusted; Model 2 was adjusted for age and gender; Model 3 was adjusted for age, gender and weight. **(a)** RCS analysis in NHANES data for Model 1. **(b)** RCS analysis in NHANES data for Model 2. **(c)** RCS analysis in clinical data for Model 1. **(d)** RCS analysis in clinical data for Model 2.

We also used RCS with four knots at 5th, 35th, 65^th^, and 95th centiles to flexibly model and visualize the relation of predicted WHtR with OSA risk in the models mentioned above based on both NHANES and clinical data. The risk of OSA was relatively flat until around 0.589 of the predicted WHtR in NHANES data (Figures 3a, b) while 0.523 of the predicted WHtR in clinical data (Figures 3c, d) and then started to increase rapidly afterwards. Above 0.589, the OR was significantly higher continuously based on the NHANES data, while above 0.523, the OR showed a trend of first increasing and then slightly decreasing with the increase of WHtR. However, overall, compared with the interval below the threshold, the OR of OSA risk was increased, indicating a strong non-linear association between WHtR and OSA risk (*p* for non-linear <0.001). Whether in NHANES data or clinical data, this nonlinear relationship remains essentially unchanged after adjusting for confounding factors (Figure 3).

### 3.3 ROC analysis of WHtR's predictive power in OSA

**Figure 4:**
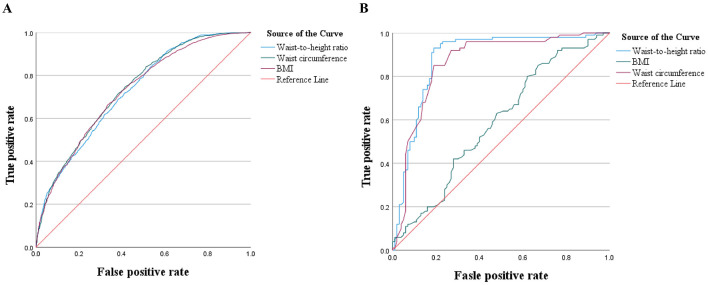
ROC analysis of WHtR's predictive power in OSA. The *y*-axis means the true positive rate (sensitivity) while the *x*-axis means the false positive rate (1-specificity). **(A)** ROC curve analysis from the NHANES data. **(B)** ROC curve analysis from the clinical data.

In both the NHANES dataset and clinical data, we conducted ROC curve analyses to assess the predictive value of WHtR, waist circumference, and BMI for OSA risk. The results indicated that in the NHANES data, the AUCs for predicting OSA risk with WHtR, waist circumference, and BMI were 0.727 ± 0.008 (95% CI, 0.711, 0.743), 0.736 ± 0.008 (95% CI, 0.720, 0.752), and 0.728 ± 0.008 (95% CI, 0.712, 0.744), respectively. Pairwise comparison using DeLong's test showed that waist circumference had a statistically significantly higher AUC than WHtR (*P* = 0.014); however, the absolute difference was minimal and unlikely to be clinically meaningful. No significant difference was observed between WHtR and BMI (*P* = 0.810) (Figure 4A) (Supplementary Table 1).

In the clinical validation group, the AUCs for WHtR, waist circumference, and BMI were 0.883 ± 0.026 (95% CI, 0.831, 0.934), 0.863 ± 0.028 (95% CI, 0.808, 0.917), and 0.588 ± 0.040 (95% CI, 0.509, 0.667), respectively (Figure 4B) (Supplementary Table 1). DeLong's test confirmed that WHtR's AUC was significantly larger than that of BMI (Z = 7.186, *P* < 0.001), while no significant difference was detected between WHtR and waist circumference (Z = 0.543, *P* = 0.587).

To further evaluate the clinical utility of WHtR, we calculated positive predictive value (PPV), negative predictive value (NPV), positive likelihood ratio (+LR), and negative likelihood ratio (-LR) at the optimal cutoff determined by Youden's index (Supplementary Table 1). In the clinical validation group, WHtR (cutoff 0.501) demonstrated excellent clinical utility with a sensitivity of 93.0%, specificity of 81.0%, PPV of 83.0%, NPV of 92.0%, +LR of 4.894, and -LR of 0.086. In contrast, BMI (cutoff 23.8 kg/m^2^) showed poor specificity (33.0%) and consequently low PPV (55.9%) and high NLR (0.454), despite comparable sensitivity. These metrics underscore that WHtR provides superior discrimination of true OSA cases from non-cases in a clinically referred population with high pre-test probability.

### 3.4 Sensitivity analysis: subgroup analysis by sex and age in the clinical data

**Figure 5:**
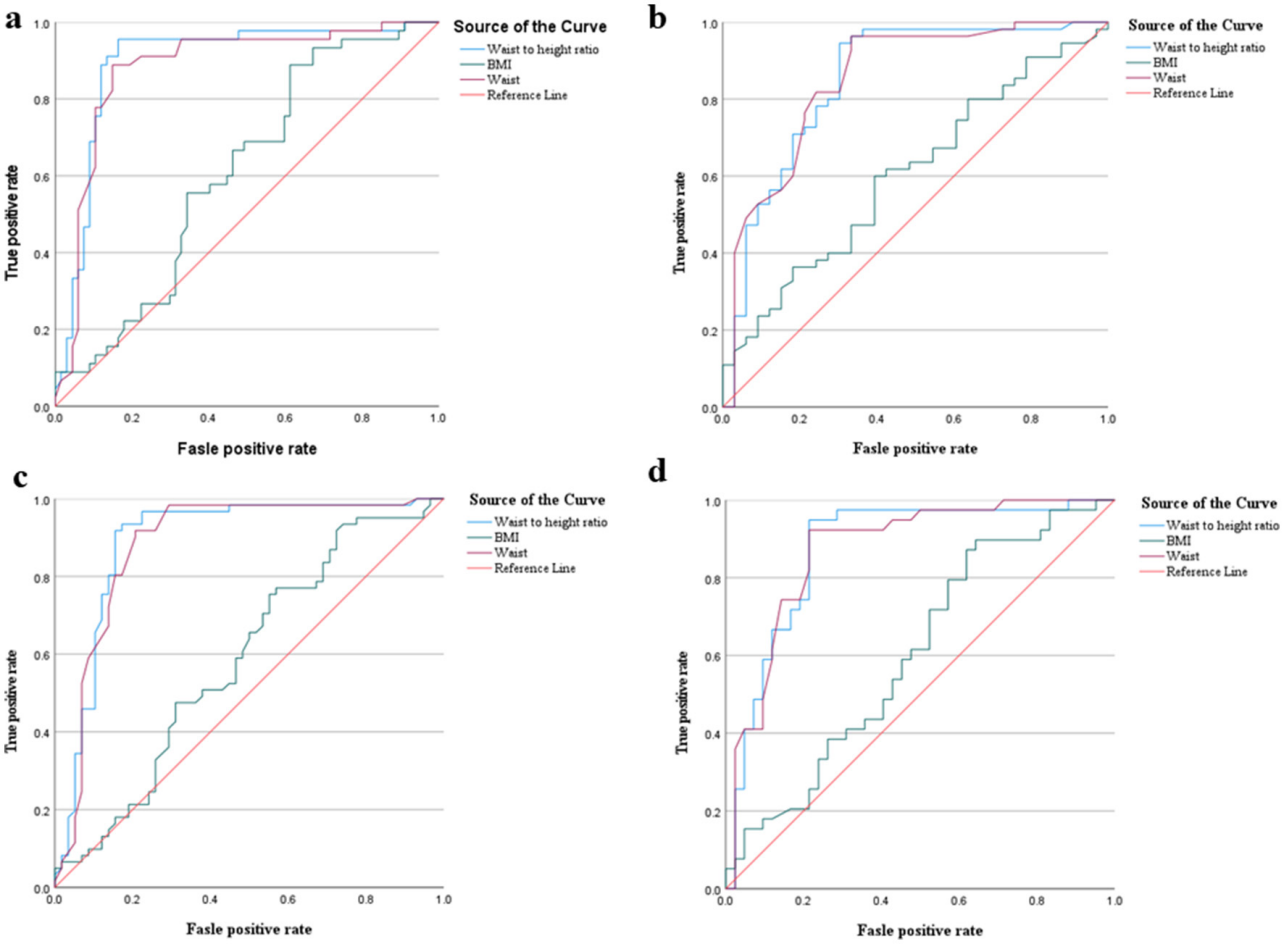
ROC analysis of WHtR's predictive power in the subgroup of clinical setting. The y-axis means the true positive rate (sensitivity) while the *x*-axis means the false positive rate (1-specificity). **(a)** ROC curve analysis in the group of age <60. **(b)** ROC curve analysis in the group of age ≥60. **(c)** ROC curve analysis in the male group. **(d)** ROC curve analysis in the female group.

Population characteristics of the subgroup subjects was described in Table 3. And the predictive performance of WHtR, waist circumference, and BMI was further evaluated in subgroups of the clinical data (Table 4, Figure 5). Among both males (*n* = 119) and females (*n* = 81), WHtR maintained the highest AUC (males: 0.890, 95% CI 0.824, 0.957; females: 0.875, 95% CI 0.793, 0.956) for predicting OSA, consistently surpassing that of BMI (males: 0.587; females: 0.602) and comparable to or slightly better than waist circumference. DeLong's test confirmed that WHtR's AUC was significantly larger than that of BMI in both genders (males: *p* < 0.001; females: *p* = 0.007). Similarly, when stratified by the age of 60, WHtR retained the higher predictive value in both the younger (<60 years) and older (≥60 years) subgroups (AUCs: 0.896 and 0.849, respectively), with its superiority over BMI remaining statistically significant (all *p* < 0.001). These sensitivity analyses confirm that the superior predictive value of WHtR for OSA is robust across key demographic subgroups in clinical settings.

**Table 3:** Population characteristics of sub-groups in clinical data.

Variable	OSA	non-OSA	Statistics	*P* value
Age <60	*n* = 45	*n* = 67		
Waist circumference (cm)	96.00 (92.00, 101.50)	79.00 (75.00, 83.80)	−6.804	<0.001^*^
BMI (Kg/m^2^)	29.13 (25.10, 31.81)	26.42 (22.76, 31.56)	−1.941	0.052^*^
Waist-to-height ratio	0.56 (0.52, 0.61)	0.46 (0.45, 0.47)	−7.902	<0.001^*^
Age ≥60	*n* = 55	*n* = 33		
Waist circumference (cm)	95.00 (89.00, 100.00)	77.70 (75.00, 86.50)	−5.523	<0.001^*^
BMI (Kg/m^2^)	26.67 (24.12, 30.46)	25.51 (23.09, 27.61)	−1.638	0.101^*^
Waist-to-height ratio	0.56 (0.53, 0.61)	0.46 (0.45, 0.53)	−5.465	<0.001^*^
Male	*n* = 61	*n* = 58		
Waist circumference (cm)	97.00 (92.00, 104.00)	80.55 (77.92, 86.00)	−7.209	<0.001^*^
BMI (Kg/m^2^)	28.22 (26.03, 30.96)	26.92 (24.15, 31.20)	−1.630	0.103^*^
Waist-to-height ratio	0.56 (0.53, 0.61)	0.46 (0.45, 0.48)	−7.345	<0.001^*^
Female	*n* = 39	*n* = 42		
Waist circumference (cm)	90.00 (85.00, 100.00)	74.75 (72.65, 80.32)	−5.774	<0.001^*^
BMI (Kg/m^2^)	25.20 (23.23, 30.81)	24.19 (22.11, 29.13)	−1.579	0.114^*^
Waist-to-height ratio	0.57 (0.52, 0.62)	0.46 (0.45, 0.48)	−5.804	<0.001^*^

OSA, Obstructive sleep apnea. ^*^Mann–Whitney U test for continuous variables.

**Table 4:** Subgroup analysis of the predictive value by sex and age in the clinical—data.

Subgroup	Variable	Area under the curve	*P*	95% CI		Cut-off	Sensitivity	Specificity
Age <60 (*n* = 112)	Waist to height ratio	0.896	<0.001	0.830	0.962	0.484	0.956	0.836
	BMI (kg/m2)	0.608	0.052	0.504	0.713	24.428	0.889	0.388
	Waist circumference (cm)	0.880	<0.001	0.810	0.950	86.500	0.889	0.851
Age ≥60 (*n* = 88)	Waist to height ratio	0.849	<0.001	0.758	0.941	0.502	0.945	0.697
	BMI (kg/m2)	0.605	0.102	0.485	0.725	25.989	0.600	0.606
	Waist circumference (cm)	0.853	<0.001	0.765	0.940	81.750	0.964	0.667
Male (*n* = 119)	Waist to height ratio	0.890	<0.001	0.824	0.957	0.506	0.918	0.845
	BMI (kg/m2)	0.587	0.103	0.483	0.690	26.092	0.754	0.448
	Waist circumference (cm)	0.883	<0.001	0.815	0.951	86.500	0.918	0.793
Female (*n* = 81)	Waist to height ratio	0.875	<0.001	0.793	0.956	0.484	0.949	0.786
	BMI (kg/m2)	0.602	0.114	0.478	0.726	22.541	0.897	0.357
	Waist circumference (cm)	0.873	<0.001	0.793	0.952	81.650	0.923	0.786

## 4 Discussion

OSA is a common condition, and its routine diagnosis primarily relies on PSG. However, the accessibility of this examination is relatively low, and currently, various screening methods (such as questionnaires, portable sleep monitors) are used clinically, which can partially make up for this deficiency. The commonly used scoring criteria in questionnaires are based on BMI, but BMI cannot fully reflect obesity or the risk of OSA. Therefore, we conducted research on the relationship between WHtR and the risk of OSA, attempting to find a more suitable tool for assessing the risk of OSA.

We conducted a comparative analysis of three anthropometric indicators—WHtR, waist circumference, and BMI—to assess their association with the risk of OSA in the American population, and we verified this by conducting a retrospective analysis of case data from a sleep center in China. Firstly, we conducted a differential test on WHtR, waist circumference, and BMI between the OSA group and the non-OSA group from the NHANES database, finding significant differences in these three indicators between the two groups. Then, logistic regression analysis was performed for each of these indicators and the risk of OSA, suggesting that the OR value for WHtR (2.780) was greater than for waist circumference (1.060) and BMI (1.155), with significant statistical differences (*p* < 0.001). Notably, the effect size observed in the clinical validation group (OR = 11.998 per 0.1 unit WHtR) was substantially larger than that in the NHANES (OR = 2.780 per 0.1 unit). Subsequently, we further explored the dose-response relationship between WHtR and OSA risk using RCS. It was confirmed that after WHtR exceeded the threshold value of 0.589, the risk of OSA increased rapidly with the rise of WHtR. Finally, the predictive value of these three indicators for OSA risk was assessed using ROC curve analysis. DeLong's test showed that waist circumference had a statistically significantly higher AUC than WHtR, however, the absolute difference was minimal and unlikely to be clinically meaningful. No significant difference was observed between WHtR and BMI. To enhance the reliability and statistical power of our results, we verified the aforementioned findings using clinical case data. The ROC analysis indicated that WHtR had the strongest predictive value for OSA risk and the optimal WHtR cut-off identified in clinical data (0.501) was less than that in NHANES data (0.552). This six-fold difference of OR and the discrepancy of cut-off value in NHANES data and clinical data maybe expected for several reasons. First, the clinical database employed PSG, the gold standard for OSA diagnosis, whereas NHANES relied on self-reported questionnaire data. Non differential misclassification of disease status in NHANES would be expected to bias the observed OR toward the null value, resulting in underestimation of the true association and shift in the optimal cut-off. Second, the clinical study used a 1:1 case-control design that maximized contrast between OSA cases and controls, whereas NHANES was a cross-sectional survey of the general population with lower disease prevalence. Third, the clinical sample comprised referred patients with high pre-test probability of OSA, who likely represented a more homogeneous and severe phenotype compared to the community-dwelling NHANES participants. Fourth, measurement protocols differed. NHANES used standardized health technician measurements in mobile examination centers, while the clinical study used bedside measurements by sleep specialists, which may introduce systematic differences in waist circumference assessment. Fifth, ethnic differences in body composition and OSA susceptibility between US (mixed ethnicity) and Chinese populations may affect the optimal anthropometric thresholds. In summary, the overall trends from the analyses of both datasets are consistent, demonstrating a stronger association of WHtR with OSA risk. The magnitude of these differences reflects not only measurement and diagnostic ascertainment differences but also the distinct clinical contexts: population-based screening vs. tertiary referral center evaluation. These findings underscore that WHtR cut-offs are population-specific and require external validation before application in different clinical or geographic settings.

Traditionally, BMI has been considered a key marker of obesity, and previous studies have typically focused on the relationship between BMI and the risk of OSA. Some studies have found that there is a positive correlation between BMI and the risk of OSA (22, 23); for each unit increase in BMI, the Apnea-Hypopnea Index (AHI) increases by 14% (23). However, there is also research suggesting that the association between BMI and OSA risk may be influenced by gender, and that BMI is not the best predictor of OSA risk in women (24). Some studies have focused on women with OSA, suggesting that both pre- and post-menopausal women, WHtR and waist circumference are the most closely related indicators to OSA, with waist circumference being particularly strongly associated with the severity of OSA in post-menopausal women (25). Our study results suggest that an increase in BMI is associated with an increased risk of OSA, but its correlation and predictive value for OSA are inferior to those of WHtR in the clinical data. Since the NHANES database does not contain statistical data for AHI, we did not conduct statistical analysis on the severity of AHI in relation to BMI and WHtR. Instead, we focused more on analyzing the predictive value of these three obesity-related indicators for the risk of OSA. Previous studies have already proposed the correlation between BMI, WHtR, and waist circumference with OSA, but they have not compared the correlation and predictive value of these indicators (26). This is the basis for our choice of these three indicators, and we believe that among them, WHtR has the strongest correlation with OSA risk and the best predictive value in the clinical validation group, which also complements previous research. Additionally, some researchers have focused on individuals with OSA who also have central obesity, suggesting that the combined use of BMI and WHtR has a better predictive value for OSA risk (AUC: 0.627) compared to using BMI alone (AUC: 0.626) or WHtR alone (AUC: 0.584) (27). In contrast, our study did not define the degree or type of obesity in the subjects from either the NHANES dataset or clinical cases.

WHtR, which is the ratio of waist circumference to height, precisely quantifies central obesity, that is, the degree of abdominal fat accumulation (12). Previous studies have shown that a critical value of 0.5 for WHtR can be applied to different genders and ethnic groups and is generally considered a universal threshold for central obesity in children (age ≥six) and adults (12). In a study of children aged 3 to 11 years, WHtR was confirmed to have the ability to distinguish between OSA and primary snoring (28). In adults who snore, male gender and a WHtR of ≥0.55 were good predictors for moderate to severe OSA (29). These studies all coincide with the findings of my research. In addition, in the context of cardiovascular diseases, WHtR also serves as a good predictive indicator. Adult meta-analyses indicate that WHtR is equal to or slightly superior to waist circumference in predicting higher cardiometabolic risk, and superior to BMI (30). Pasdar and colleagues also reached similar conclusions, suggesting that WHtR can better predict cardiovascular events in the obese population compared to waist circumference and BMI (14). Not only that, but also Sun and others used the NHANES database to study the relationship between anthropometric indicators such as BMI, waist circumference, and WHtR with the prevalence and mortality of OSA. They proposed that both BMI and waist circumference are positively correlated with the prevalence of OSA and negatively correlated with mortality; WHtR is positively correlated with both the prevalence and mortality of OSA (31). In a prospective study in the United States, WHtR was found to be a better predictor of early mortality than BMI. Among individuals aged 12–39 years, compared to the group with WHtR less than 0.5, those with WHtR greater than 0.65 had a 139% higher risk of death before the age of 55 (32). These findings underscore the importance of controlling obesity in young people, particularly the early detection and intervention of central obesity. It is evident that the importance of WHtR cannot be overlooked, it is necessary to observe the relationship between WHtR and OSA in more prospective studies. Further exploration of the underlying mechanisms should be emphasized.

This study compares WHtR with the traditional obesity indicator BMI, providing valuable insights into the relationship and predictive value of WHtR with OSA risk. It offers some basis for whether WHtR can replace BMI in screening questionnaires as a scoring criterion. The relationship of WHtR and OSA risk can be attributed to its direct reflection of central obesity, particularly visceral adiposity. Unlike BMI, which measures overall body mass without distinguishing between fat, muscle, or its distribution, WHtR specifically captures abdominal fat accumulation. This is physiologically critical for OSA pathogenesis through several interconnected mechanisms. First, visceral fat is metabolically active, secreting pro-inflammatory cytokines (e.g., TNF-α, IL-6) and adipokines that may promote systemic inflammation and autonomic dysfunction result in the aggravation of OSA (33). Second, abdominal fat reduces the lung volume and tracheal traction of pharyngeal tissues. This leads to a smaller, more collapsible pharyngeal airway (34). Third, the pharynx is a tubular cavity lacking bony and cartilaginous support, and its anatomical characteristics determine that it has high compliance and collapsibility. Fat deposition in the neck region, which correlates with abdominal obesity, directly narrows the upper airway lumen and increases tissue pressure surrounding it, predisposing to collapse during sleep (35, 36). Therefore, WHtR serves as an integrated, clinically accessible metric that encapsulates these key pathophysiological pathways, which making it a more direct and sensitive indicator of OSA risk than BMI.

Beyond establishing the relationship between WHtR and OSA risk, a critical question for clinical translation is how this anthropometric measure can be integrated into existing OSA screening workflows. Whether and how WHtR should replace or supplement BMI in existing screening questionnaires such as STOP-Bang and NoSAS remains to be determined. The optimal integration strategy, whether as a direct substitute, an additional item, or a weighted scoring component which requires prospective head-to-head comparisons. Beyond questionnaire integration, the translation of WHtR into clinical practice requires parallel efforts in implementation research. Routine WHtR measurement in primary care settings is feasible with minimal training and equipment. Future studies should evaluate whether this simplicity translates into improved screening adherence, earlier detection of high-risk individuals, and ultimately, higher referral rates for polysomnography.

However, several limitations of this study should be strictly acknowledged. Firstly, the most significant limitation of this study is the absence of polysomnography for OSA diagnosis in NHANES data study. OSA status in NHANES was determined through self-reported questionnaires, which introduces non-differential misclassification of disease status. According to established epidemiological principles described in Modern Epidemiology (37), independent non differential misclassification of a binary disease status is predictable in direction which toward the null value. This bias would be expected to attenuate the observed odds ratio, potentially resulting in an underestimate of the true association between WHtR and OSA risk. Non-differential misclassification typically reduces the apparent discriminative ability of a predictive model, resulting in underestimation of the true AUC (38). Given that our observed AUC was 0.727±0.008 in NHANES data, the true discriminative performance of WHtR for OSA prediction is likely higher. However, without internal validation data linking questionnaire responses to PSG results in the NHANES population, we cannot provide a precise corrected AUC estimate. The integration of objective sleep monitoring data in population-based studies would significantly strengthen the evidence base for using WHtR as a screening tool for OSA risk. Secondly, the analysis based on the NHANES database is a cross-sectional study, which does not allow for causal inference, and this is a survey data from the American population, and further surveys in other regions and ethnicities are needed to verify to ensure broader applicability. Lastly, although we randomly selected cases from the sleep center who completed PSG examinations in 2024 for a retrospective analysis to verify our previous conclusions, the sample size of clinical case data is relatively small, and they may be influenced by unnoticed, unmeasured biases or confounding factors. To evaluate whether the achieved sample size provided adequate statistical precision, we conducted a *post-hoc* precision analysis for the primary diagnostic accuracy outcomes. *Post-hoc* analysis demonstrated that the achieved 95% confidence interval widths were ± 6.5% for sensitivity (observed 93.0%, 95% CI: 86.5–97.4%), ± 9.0% for specificity (observed 81.0%, 95% CI: 72.0–88.0%), and ± 0.052 for AUC (observed 0.883, 95% CI: 0.831–0.934), all meeting or exceeding our pre-specified precision targets of ± 10% and ± 0.05, respectively (Supplementary Table 2). However, we acknowledge that this sample size limits the precision of subgroup analyses. Future larger multi-center studies are warranted to validate these findings. Moreover, the prospective studies are needed to validate the predictive value of WHtR in diverse populations and to establish universally applicable WHtR cut-offs or develop population-specific reference values tailored to local epidemiological and healthcare contexts.

## 5 Conclusion

In conclusion, our findings from two independent datasets indicate that WHtR is a strong indicator of increased OSA risk. WHtR demonstrates superior predictive value compared to BMI in clinically referred populations, which shows promise as an accessible screening tool in clinical settings, while its utility in population-based screening requires further validation.

## Data Availability

Publicly available datasets were analyzed in this study. This data can be found here: The datasets generated and/or analyzed during the current study are available in NHANES (https://wwwn.cdc.gov/nchs/nhanes/Default.aspx). Further inquiries can be directed to the corresponding authors.
