# Supplementary material for: Waist-to-height ratio for OSA risk: a comparative analysis of NHANES and a clinical data
Source: Front Med (Lausanne). 2026 May 29;13:1842979. doi: 10.3389/fmed.2026.1842979 (PMC13260219; doi:10.3389/fmed.2026.1842979)
Supplement: Supplementary file 1 [file Table_1.docx]

| **Supplementary table 1. Diagnostic performance metrics and statistical comparisons of WHtR, waist circumference, and BMI for OSA prediction** | | | | | | | | | | | | | |
| --- | --- | --- | --- | --- | --- | --- | --- | --- | --- | --- | --- | --- | --- |
| **Dataset** | **Variable** | **AUC** | ***P*** | **95% CI** | **Delong test (vs. WHtR (Z, *P*))** | **Cut-off** | **Sensitivity** | **Specificity** | **PPV** | **NPV** | **PLR** | **NLR** |  |
| NHANES  (n=3715) | Waist to height ratio | 0.727 | 0.008 | 0.711-0.743 | - | 0.552 | 0.839 | 0.468 | 0.571 | 0.772 | 1.577 | 0.344 |  |
|  | BMI（kg/m^2^） | 0.728 | 0.008 | 0.712-0.744 | 0.241,0.810 | 28.450 | 0.661 | 0.668 | 0.625 | 0.699 | 1.994 | 0.506 |  |
|  | Waist circumference(cm) | 0.736 | 0.008 | 0.720-0.752 | 2.452,0.014 | 97.050 | 0.714 | 0.618 | 0.850 | 0.788 | 1.865 | 0.463 |  |
| Clinical  (n=200) | Waist to height ratio | 0.883 | <0.001 | 0.831-0.934 | - | 0.501 | 0.930 | 0.810 | 0.830 | 0.920 | 4.894 | 0.086 |  |
|  | BMI（kg/m^2^） | 0.588 | 0.029 | 0.509-0.667 | -7.186,<0.001 | 23.850 | 0.850 | 0.330 | 0.559 | 0.687 | 1.268 | 0.454 |  |
|  | Waist circumference(cm) | 0.863 | <0.001 | 0.808-0.917 | -0.543,0.587 | 86.500 | 0.850 | 0.810 | 0.825 | 0.845 | 4.473 | 0.185 |  |
