# Supplementary material for: Waist-to-height ratio for OSA risk: a comparative analysis of NHANES and a clinical data
Source: Front Med (Lausanne). 2026 May 29;13:1842979. doi: 10.3389/fmed.2026.1842979 (PMC13260219; doi:10.3389/fmed.2026.1842979)
Supplement: Supplementary file 2 [file Table_2.docx]

| **Supplementary table 2. Post-hoc precision analysis for WHtR to OSA in clinical data.** | | | | |
| --- | --- | --- | --- | --- |
| Parameter | Observed Value | 95% CI | Precision Achieved | Target |
| Sensitivity | 93.0% | 86.5–97.4% | ±6.5% | ±10% |
| Specificity | 81.0% | 72.0–88.0% | ±9.0% | ±10% |
| AUC | 0.883 | 0.831–0.934 | ±0.052 | ±0.05 |

The 95% confidence intervals were calculated using standard methods (Clopper-Pearson method for sensitivity and specificity). WHtR, Waist-to-height ratio; OSA, Obstructive sleep apnea. AUC, Area under the curve.
